# Is androgen production in association with immune system activation potential evidence for existence of a functional adrenal/ovarian autoimmune system in women?

**DOI:** 10.1186/1477-7827-11-58

**Published:** 2013-06-27

**Authors:** Norbert Gleicher, Andrea Weghofer, Vitaly A Kushnir, Aya Shohat-Tal, Emanuela Lazzaroni, Ho-Joon Lee, David H Barad

**Affiliations:** 1 Center for Human Reproduction, New York, NY 10021, USA; 2 Foundation for Reproductive Medicine, New York, NY 10021, USA; 3 Department of Gynecologic Endocrinology and Reproductive Medicine, Medical University Vienna, Vienna 1090, Austria

**Keywords:** Androgens, Testosterone, Ovarian Reserve, Autoimmunity, In Vitro Fertilization

## Abstract

**Background:**

Low functional ovarian reserve (FOR) is at all ages associated with low testosterone (T) levels. Causes are, however, unknown. We, therefore, investigate whether androgens with low FOR are associated with non-specific immune system activation.

**Methods:**

322 infertile women with low and normal FOR (controls) were assessed with a broadly based immune profile, which in previous studies has proven effective in differentiating infertile patients with and without immune system activation. Patients were either immune-positive (greater than or equal to one positive tested parameter) or immune negative (no positive test). 135 suffered from prematurely diminished FOR (POA/OPOI; < age 38), 155 from physiologic diminished FOR due to age (DOR; > age 40), and 32 were controls (< age 38 with normal age-specific FOR). Prevalence of immune-positive vs. negative was assessed in all 3 patient groups.

**Results:**

Women with immune abnormalities, overall, demonstrated higher total T (TT, P = 0.004) and free T (FT, P < 0.001) levels than those without. The three clinical and two immunologic-defined patient groups demonstrated significant statistical interaction in mean TT (P = 0.008), with mean TT and FT in women with positive immune findings being significantly higher in control than in POA/OPOI and physiologic DOR patients (all 4 differences P < 0.001). No such differences between the three groups were seen in women without immune abnormalities.

**Conclusions:**

In this study we used a definition of immune-positivity, which favors sensitivity over specificity, resulting in significant numbers of false-positives but likely only few false-negatives. The study allows suggesting the possibility of an immune system-derived androgen-production factor (APF), which maintains normal androgen levels but is deficient in women with low FOR and immune system inactivity. Existence of such an APF would suggest the presence of a still unknown functional adrenal autoimmune system.

## Background

What in a woman constitutes total ovarian reserve (TOR) has remained poorly defined [1,2]. Most investigators describe ovarian reserve (OR) as the sum of all remaining follicles. This definition, however, includes in a majority the non-growing pool of primordial follicles, which cannot be reliably assessed [1,2]. Because total primordial follicles appear to correlate to recruited follicles, and because anti-Müllerian hormone (AMH) appears representative of small growing follicles (after recruitment), AMH is now widely considered the most accurate tool to assess TOR [1,3]. We have come to call the pool of small growing follicles the functional ovarian reserve (FOR) because it best reflects what, a few months later, can be expected as oocyte yield in an in vitro fertilization (IVF) cycle [2].

Small growing follicles in mouse studies have been demonstrated dependent on androgen receptor (AR)-mediated effects on granulosa cells [4], which, synergistic with follicle stimulating hormone (FSH), lead to normal follicle growth and oocyte development. Interruption of AR effects in granulosa cells, but not oocytes, leads to various infertility-associated conditions [4]. Androgen sources are adjacent theca cells in the ovary and the zona reticularis of the adrenals.

That androgens at these stages of follicle development may also be important in humans has been suggested by observations in association with IVF [5,6] and by reported improvements in fertility treatment outcomes following supplementation of women at all ages with low FOR with dehydroepiandrosterone (DHEA) [7]. Indeed, the latter improvements have been demonstrated to correlate well with improvements of testosterone levels after DHEA supplementation [8].

Based on these observations, we developed the hypothesis that low FOR may represent an androgen deficiency state, and recently presented confirmatory evidence [9]. Androgen deficiency is more profound in younger women with prematurely diminished FOR (DFOR), i.e. premature ovarian aging (POA), by others given the acronym occult primary ovarian insufficiency (OPOI). The same study also offered preliminary evidence that concomitantly low cortisol levels may accompany androgen insufficiency. Low androgen in association with DFOR may, therefore, be adrenal as well as ovarian in etiology, mimicking in opposing ways the hyperandrogenism and excessive follicle production in some women with polycystic ovary syndrome (PCOS) [9].

It, therefore, is tempting to hypothesize that PCOS and POA/OCPOI reflect functionally opposing clinical conditions of adrenals and ovaries in combination, similar to hypo- and hyperthyroidism. Hypothyroidism is usually the consequence of Hashimoto’s thyroiditis, associated with anti-thyroglobulin and anti-thyroid peroxidase antibodies [10], while autoantibodies to the thyrotropin receptor result in hyperthyroidism [11]. Functional autoantibodies in autoimmunity no longer are only limited to autoimmune endocrine diseases [12]. They, therefore, appear more common in autoimmunity than previously assumed [13].

We in the past searched for but failed [14] or found only statistically marginal data [15] in support of autoimmunity in association with POA (OPOI). Those studies, however, did not consider the patients’ androgen status. The recently recognized importance of hypoandrogenism in defining DFOR [9], now suggests that androgen levels may be associated with immune system activation. As this study will demonstrate, immune system activation, indeed, appears to be associated with androgen levels.

## Methods

### Study populations

This study involved the retrospective investigation of a cohort of 322 infertile women for whom complete data sets were available in the center’s electronic research data bank of The Center for Human Reproduction – New York, a private clinical and research center, specializing in reproductive endocrinology and infertility. They were divided into three distinct groups: (i) 135 Women with POA/OPOI (Group 1), defined as patients under age 38 years with abnormally elevated age-specific FSH [16] and/or abnormally low age-specific AMH [17]; (ii) 155 Women above age 40 years with age-dependent DFOR, here simply described as physiologic DOR (Group 2); and (iii) 32 control patients, defined as infertility patients under age 38 with normal age-specific FOR, based on FSH and AMH levels (controls). To create a clear statistical distinction between POA/OPOI and DOR patients, women between ages 38 and 40 years were excluded from this study.

### Definition of immune system activation

Because of the reported high prevalence of autoimmune abnormalities in infertile women and associated miscarriage risks [18,19], our center obtains an (auto)immune panel in the initial investigation of every newly presenting female infertility patient. Such a panel includes the following: Antinuclear antibody, anti-phospholipid antibodies (lupus anticoagulant, anti-cardiolipin, anti-phosphatidylserine, β2-glycoprotein, for IgG, IgM and IgA isotypes), anti-thyroid antibodies (anti-thyroglobulin, anti-thyroid peroxidase), anti-adrenal antibody (21-hydroxylase) and anti-ovarian antibodies (non-specific) as well as total immunoglobulins in IgG, IgM, IgA and IgE.

The principal rationale for this panel is not the diagnosis of specific autoimmune conditions but the detection of an activated autoimmune system, which may suggest the presence of a polyclonal immune activation at a sub-clinical level of autoimmune activity. Even with only a single positive result on any one of above described tests, a patient is, therefore, considered “affected.”

We fully recognize the non-specific nature of this definition of immune system activation, and the high rate of expected false-positive patients it generates. This kind of analysis, however, has proven itself in the past in a number of studies [14,15,20-22] and, likely, to an acceptable degree, precludes presence of autoimmunity in absence of any detected laboratory abnormalities. We, thus, assume relatively satisfactory negative predictability for immune system activation from our immune screen, though at the expense of a considerable degree of false positive results.

**Figure 1:**
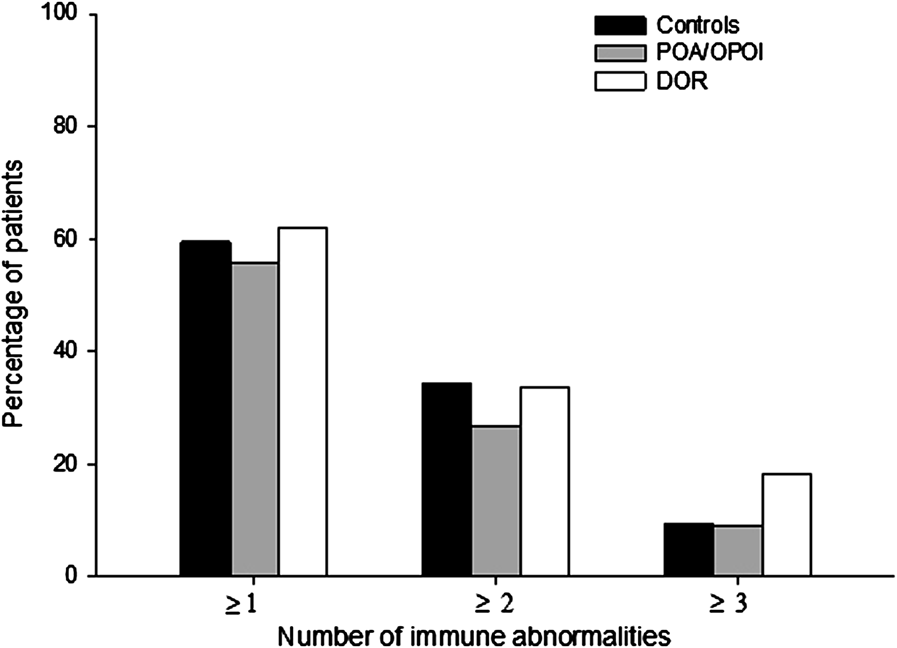
**Prevalence of immune abnormalities in investigated patient groups.** X-axis demonstrates the number of immune abnormalities per patient (range 1–3). The number of immune abnormalities detected in in controls, POA/OPOI and DOR patients did not differ. Women with DOR, however, demonstrated a trend toward more ≥3 abnormalities in comparison to controls and POA/OPOI patients (P = 0.06), with up to 9 percent difference.

Utilizing these criteria 59.4 percent of control patients, 55.6 percent of POA/OPOI (Group 1) and 61.9 percent of physiologic DOR (Group 2) patients were defined as immunologically positive, a non-significant difference in prevalence (P = 0.54). Furthermore, 34.4 percent of controls, 26.7 percent of Group 1 and 33.5 percent of women Group 2 patients (P = 0.40) demonstrated two or more immune abnormalities, 9.4 percent of controls, 8.9 percent of POA/OPOI and 18.1 percent of physiologic DOR patients demonstrated three or more immune abnormalities (P = 0.06) (Figure 1). Prevalence of immune abnormalities, thus, did not differ between controls and study groups, though, not surprisingly considering their older age, women with physiologic DOR (Group 2) did demonstrate a trend towards more autoimmune abnormalities in comparison to the other two, younger patient groups, with up to a 9 percent difference.

All tests were performed by commercial laboratory testing in accordance with patient preference and insurance requirements. A test was considered positive if the result fell outside of normal range, as defined by the processing laboratory.

### Androgen determinations

Androgen testing is also a routine part of every patient’s initial evaluation and includes dehydroepiandrosterone (DHEA), DHEA-sulfate (DHEAS), androstenedion, free testosterone (FT), and total testosterone (TT). These tests were also performed by commercial laboratories [9].

### Statistical analyses

The Mann–Whitney U and ANOVA tests were used for a majority of statistical analyses. All post hoc procedures utilized the Holm Sidak test to determine significance. Differences in distributions between categorical data were compared using chi-square tests. All tests were 2-tailed, with P < 0.05 considered statistically significant.

### Institutional Review Board (IRB)

Since this study involved only the retrospective review of anonymized electronic medical records in the center’s research database, it required only expedited IRB review, which was obtained. Our center’s patients at time of initial consultation sign an informed consent, which permits the use of medical record data for research purposes, as long as anonymity of patient identity and confidentiality of the medical record is maintained. Research personnel are also committed in writing to confidentiality under federal HIPAA rules.

## Results

Table 1 summarizes patient characteristics of all three study groups. As can be seen, mean ages were in controls 33.1 ± 3.7 years, in POA/OPOI women 34.5 ± 3.2 and in physiologic DOR, 43.2 ± 2.4 years (Sidak test, controls vs. DOR and POA/OPOI vs. DOR, both P < 0.001; controls vs. POA/OPOI P = 0.04). The groups did not differ in body mass index (BMI).

**Table 1:** Patient characteristics of controls, POA/OPOI and DOR patients

	**POA/OPOI**	**DOR**	**Controls**	**P value (Sidak test)**
N	135	155	32	
Age (years)	34.5 ± 3.2^1,2^	43.2 ± 2.4^1,3^	33.1 ± 3.7^2,3^	^1^0.001, ^2^0.04, ^3^0.001
BMI (kg/m^2^)	24.0 ± 4.8	24.3 ± 5.0	22.0 ± 5.5	
FSH (mIU/mL)	13.4 ± 14.2^4,5^	17.8 ± 13.1^4,6^	6.3 ± 2.5^5,6^	^4^0.02, ^5^0.02, ^6^0.001
AMH (ng/mL)	0.9 ± 1.1^7,8^	0.3 ± 0.3^7,9^	3.3 ± 2.1^8,9^	^7^0.001, ^8^0.001, ^9^0.001
***Race *n (%)**				
*Caucasian*	90 (66.7)	119 (76.8)	19 (59.4)	
*African*	16 (11.9)	18 (11.6)	4 (12.5)	
*Asian*	29 (21.5)	18 (11.6)	9 (28.1)	

Values are presented as means ± SD.

They, however, did significantly vary in OR parameters: FSH was 6.3 ± 2.5 mIU/mL in controls, 13.4 ± 14.2 mIU/mL in POA/OPOI and 17.8 ± 13.1 mIU/mL in physiologic DOR patients (Sidak test, controls vs. POA/OPOI, P = 0.02; controls vs. DOR, P < 0.001; POA/ OPOI vs. DOR P = 0.02). AMH values were in controls 3.3 ± 2.1 ng/mL, in POA/OPOI 0.9 ±1.1 ng/mL and in DOR patients 0.3 ± 0.3 ng/mL (Sidak test, all 3 groups P < 0.001 in comparison to each other).

Adjusted for BMI and age, DHEA, DHEAS did not differ amongst women with or without presumed autoimmunity. Women with immune abnormalities, however, demonstrated overall higher TT and FT levels than women without such abnormalities (Sidak test, P = 0.004 and P < 0.001, respectively).

**Figure 2:**
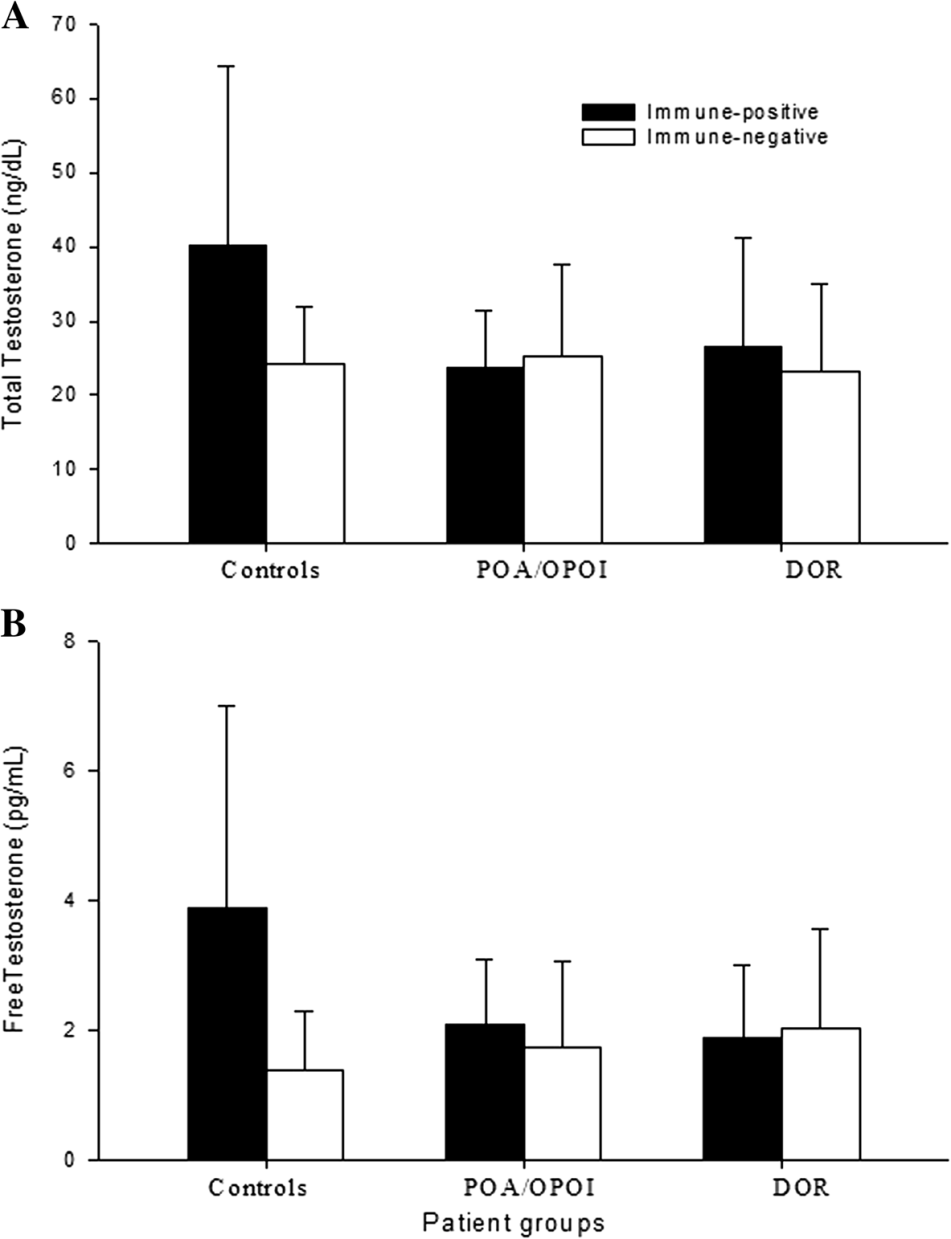
**Testosterone levels in patients with and without presumed immune activation. A** demonstrates TT and **B** FT levels; both were, overall, among women with autoimmunity significantly elevated (P = 0.004 and P < 0.001, respectively) but in women with POA/OPOI and DOR significantly diminished (both P < 0.001) in comparison to controls. Women without evidence of immune system activation, in contrast, overall, demonstrated lower androgen levels (as noted above) and did not differ in either TT or FT between controls and POA/OPOI and physiologic DOR patients.

When in each patient group women were stratified by presumed immune activation, differentiating between women with no and one or more immune abnormalities, there was a statistically significant interaction in mean TT [F (2,240) = 4.89, P = 0.008]. Post hoc comparisons, using the Sidak test, suggested that mean TT, stratified for immune activation, in women with positive autoimmunity was significantly higher in controls (M = 40.2, SD = 24.1) than in women with POA/OPOI (M = 23.6, SD = 7.8, P < 0.001) and in women with physiologic DOR (M = 26.6, SD = 14.5, P < 0.001) (Figure 2a).

The same was noted in association with mean FT levels: Interaction between three patient groups and two immunologic definitions was [F (2, 207) = 9.47, P < 0.001]; and, amongst autoimmune-positive women controls (M = 3.9, SD = 3.1) demonstrated significantly higher FT than POA/OPOI patients (M = 2.1, SD = 1.0; P < 0.001) and women with physiologic DOR (M = 1.9, SD = 1.1; P < 0.001) (Figure 2b).

These statistical differences in testosterone between control patients and both study groups were, however, not seen in women without evidence of autoimmunity (Figure 2a,b). None of the investigated immune parameters, in isolation, were statistically associated with androgen levels, including anti-adrenal and anti-ovarian antibodies.

## Discussion

This study demonstrates statistical associations between immune system activation and androgen levels in infertile women with normal FOR and absence of both in association with POA/OPOI and age-dependent DFOR. The results, however, do not necessarily demonstrate the associations we expected. Data confirmed our earlier report that women with POA/OPOI and physiologic DOR, in principle, demonstrate lower testosterone levels than control patients with normal OR [9]. The study, however, also demonstrated higher testosterone levels with than without immune system activation. Moreover, amongst women with immune system activation, androgen levels were higher in women with normal FOR (controls) than in those with DFOR (I.e., POA/OPOI and physiologic DOR). Indeed, POA/OPOI and DOR patients, whether demonstrating signs of immune activation or not, demonstrated similar androgen levels as immunologically negative controls (Figure 2).

These observations suggests that in most young women with normal FOR a degree of immune activation exists, associated with normal to mildly elevated T levels (Figure 2), while in association with low FOR, whether due to POA/OPOI or physiologic DOR, T levels are lower in absence of evidence of immune system activation. Immune system activation, thus, appears in some fashion associated with normal to mildly elevated androgens, while in absence of such immune system activation androgens plunge.

This observation may be interpreted as suggesting that immune system activation is associated with presence of an androgen-producing factor (APF), an in itself interesting observation, considering that induction of tolerance with implantation of the paternal semi-allograft, unquestionably, requires some form of maternal immune system activation. Considering the relatively small number of patients in each of the three studied patient populations, our data, however, of course, require further confirmation.

Androgens are generally believed immunosuppressive [23,24], though also have been suggested to have an immuno-modulatory effect, either immuno-enhancing or -suppressive [25]. Assuming immune system-induced increases androgen levels, then such immuno-modulatory effects of androgens may be able to feed back, and self-control androgen production by adrenals and/or ovaries.

Anti-adrenal autoimmune responses in Addison’s disease and autoimmune ovarian insufficiency are, indeed, similar in that both are IgG1 dominated and predominantly of Th 1 type [26]. Albertini, only recently, described the ovary, “as something of an immunological hotspot,” since many genes, recently implicated in ovarian aging, are associated with immune pathways [27].

Here presented data, however, further suggest that in women with POA/OPOI and physiologic DOR the immune system’s ability to produce APF is lost. As a consequence, T levels are low (Figure 2). Two possible explanations come to mind: either the production of APF is interrupted or the effects of APF are blocked. Our results more likely suggest diminution of APF production, since blockage of APF, likely, would have to involve evidence of increased immune system activities, the opposite of what was observed.

Assuming such an androgen-regulation process in adrenals and ovaries, one also has to assume the possibility of APF overproduction. Such overproduction then could be expected to result in excessive androgen levels and, hypothetically, an ovarian PCOS- like phenotype Such phenotypes, of course, can be either hyper- or normo-androgenic [28]. PCOS and DOR may, therefore, represent opposing extremes of, possibly, immune-mediated effects on adrenals and/or ovaries [9,13].

We are not the first to suggest such an autoimmune etiology for at least some cases of PCOS [29,30]. Interestingly, González et al. just recently reported that hyperandrogenism apparently exerts anti-inflammatory effects on women with PCOS [31]. Androgen effects on early stages of follicle maturation are AR-mediated [4]. AR-activation in benign prostatic hyperplasia was only recently demonstrated produce anti-inflammatory effects [32].

Our findings raise a multitude of interesting follow up questions, with the first being how an immune process, selectively, can be associated with higher androgen levels? This study does not offer an answer, but adrenals produce the majority of a woman’s DHEA, the basic substrate for T, in turn produced in ovaries [33]. As in association with PCOS [28], at least a portion of observed testosterone levels, therefore, can be expected to be consequence of adrenal processes.

Immune-driven stimulation of androgen production in the zona reticularis of adrenals is conceivable. Immune-driven endocrine processes are often antibody-mediated [34], and even organ-specific anti-glandular autoimmunity is now increasingly recognized associated with systemic autoimmune effects [35]. In association with Addison’s disease, an autoantibody to 21-hydroxylase, a cytochrome P450 enzyme, is diagnostic [36]. Autoantibodies to other adrenal steroidogenic enzymes have also been described [37,38]. The practically complete absence of anti-21-hydroxylas antibodies in here presented patients (unpublished data), however, suggests that in this study observed immune activation is distinct from Addison’s disease.

Functional autoantibodies can be either suppressive or enhancing and have also been described in non-endocrine autoimmune diseases [12]. This led us to suggest that such regulatory autoantibodies may represent a more frequent characteristic of autoimmune diseases than is generally appreciated [13]. This study now allows for the possibility of an autoantibody network, which controls androgen production by the adrenal zona reticularis of and/or the ovarian theca, representing APF.

Women are especially prone to autoimmune diseases, especially towards autoimmune endocrinopathies and combinations of multiple autoimmune endocrinopathies, autoimmunity towards the thyroid gland being the most frequent [39].

We recently reported specific genotypes and sub-genotypes of the *FMR1* gene, associated with distinct ovarian aging patterns. The so-called *het-norm/low* sub-genotype appears associated with an ovarian PCO-like phenotype at young age, which rapidly depletes follicles, leading to early DFOR often at relatively young ages [21]. Whether the early stages of PCO-like phenotype are associated with hyperandrogenism is unknown but the same sub-genotype of *FMR1* was recently also demonstrated to convert DHEA to TT less efficiently than other *FMR1* genotypes did [8]. The specific reason why women with *het-norm/low FMR1* convert so poorly is unknown but it is interesting to note that this sub-genotype is also highly associated with autoimmune risk, while its counterpart, the *het-norm/high* sub-genotype, is protective against autoimmunity [21].

In conclusion, this study reports first supportive evidence for a, possibly, immune system-associated androgen production process, likely primarily located in adrenals and/or ovaries. We hypothesize that this process, in analogy to immune processes in other endocrine organs, may be autoantibody-driven. For the interested reader a recent review on the impact of endocrine autoimmune diseases on female fertility offers additional insights [40].

## Abbreviations

AMH: Anti-Müllerian hormone; APF: Androgen-producing factor; AR: Androgen receptor; BMI: Body mass index; DFOR: Diminished functional ovarian reserve; DHEA: Dehydroepiandrosterone; DHEAS: DHEA-sulfate; DOR: Diminished ovarian reserve; FOR: Functional ovarian reserve; FMR1: Fragile X mental retardation 1 gene; FOR: Functional ovarian reserve; FSH: Follicle stimulating hormone; FT: Free testosterone; het: Heterozygous; hom: Homozygous; IVF: In vitro fertilization; norm: Normal; OPOI: Occult primary ovarian insufficiency; OR: Ovarian reserve; PCOS: Polycystic ovary syndrome; POA: Premature ovarian aging; T: Testosterone; TOR: Total ovarian reserve; TT: Total testosterone.

## Competing interests

NG and DHB are members of the Board of the Foundation for Reproductive Medicine. NG, AW and DHB received research support, lecture fees and travel support from a variety of pharmaceutical and medical device companies, none in any way related to the issues discussed in this manuscript. NG and DHB are listed as co-inventors on two, already granted U.S. user patents, which claim therapeutic benefits from DHEA supplementation in women with DFOR. Both authors are also listed on additional pending patents in regards to DHEA supplementation and on pending patents, claiming diagnostic and therapeutic benefits from the determination of CGG repeats on the *FMR1* gene. NG is owner of the Center for Human Reproduction, where this research was performed. NG is a shareholder of Fertility Nutraceutical LLC, a producer of DHEA and other fertility-related nutraceuticals. NG and DHB receive patent royalty payments from this company.

## Authors’ contributions

NG conceived of the project, and developed the study design with participation of DHB and AW, AK, MS, whose services are greatly appreciated, largely performed data accumulation and statistical analyses. NG and DHB interpreted the data and NG wrote the manuscript, with all other authors participating in the editing and revision process. All authors read and approved of the final manuscript.
